# Subclinical synovitis detected by macrophage PET, but not MRI, is related to short-term flare of clinical disease activity in early RA patients: an exploratory study

**DOI:** 10.1186/s13075-015-0770-7

**Published:** 2015-09-25

**Authors:** Yoony Y. J. Gent, Marieke M. ter Wee, Alexandre E. Voskuyl, Debby den Uyl, Nazanin Ahmadi, Cristina Dowling, Cornelis van Kuijk, Otto S. Hoekstra, Maarten Boers, Willem F. Lems, Conny J. van der Laken

**Affiliations:** Department of Rheumatology, VU University Medical Center, De Boelelaan 1117, PO Box 7057, Amsterdam, 1081 HV The Netherlands; Department of Radiology & Nuclear Medicine, VU University Medical Center, De Boelelaan 1117, Amsterdam, 1081 HV The Netherlands; Department of Rheumatology, Jan van Breemen Research Institute | Reade, Amsterdam, dr. Jan van Breemenstraat 2, Amsterdam, 1056 AB The Netherlands; Department of Clinical Epidemiology & Biostatistics, VU University Medical Center, De Boelelaan 1117, Amsterdam, 1081 HV The Netherlands

## Abstract

**Introduction:**

Residual subclinical synovitis can still be present in joints of rheumatoid arthritis (RA) patients despite clinical remission and has been linked to ongoing radiological damage. The aim of the present study was to assess subclinical synovitis by positron emission tomography (PET; macrophage tracer ^11^C-*(R)-*PK11195) in early RA patients with minimal disease activity without clinically apparent synovitis (MDA); and its relationship with clinical outcome and magnetic resonance imaging (MRI), respectively.

**Methods:**

Baseline PET and MRI of hands/wrists were performed in 25 early MDA RA patients (DAS 44 < 1.6; no tender/swollen joints) on combined DMARD therapy. PET tracer uptake (semi-quantitative score: 0–3) and MRI synovitis and bone marrow edema (OMERACT RAMRIS) were assessed in MCP, PIP and wrist joints (22 joints/patient; cumulative score).

**Results:**

Eleven of 25 patients (44 %) showed enhanced tracer uptake in ≥ 1 joint. Fourteen of these 25 (56 %) patients developed a flare within 1 year: 8/11 (73 %) with a positive, and 6/14 (43 %) with a negative PET. In the latter, in 5/6 patients flare was located outside the scan region. Median cumulative PET scores of patients with a subsequent flare in the hands or wrists were significantly higher than those of patients without a flare (1.5 [IQR 0.8–5.3] vs 0.0 [IQR 0.0–1.0], *p* = 0.04); significance was lost when all flares were considered (1.0 [IQR 0.0–4.0] vs 0.0 [IQR 0.0–1.0], *p* = 0.10). No difference in cumulative MRI scores was observed between both groups.

**Conclusions:**

Positive PET scans were found in almost half of early RA patients with MDA. Patients with a subsequent flare in hand or wrist had higher cumulative PET scores but not MRI scores, suggesting that subclinical arthritis on PET may predict clinical flare in follow-up.

## Introduction

Rheumatoid arthritis (RA) is a chronic, inflammatory disease that affects the joints. RA patients with high levels of disease activity have worse clinical and radiological outcomes than patients with minimal disease activity (MDA) or patients in remission [1]. A state of true remission [2] is the main therapeutic objective of RA, and certainly MDA [3] is becoming a more realistic goal owing to intensified treatment with disease-modifying anti-rheumatic drugs (DMARDs) [4]. We showed treat-to-target regimens such as Combinatietherapie bij Reumatoïde Artritis (COBRA) or COBRA-light therapy—comprising methotrexate (MTX) with or without sulfasalazine (SSZ) and high-dose or moderate-dose prednisolone respectively—to be effective in establishing MDA or remission, at least partly by their target design in which patients without a favorable response (clinical remission) are offered other, probably more powerful, antirheumatic drugs [5, 6]. In addition, once drug-induced remission has been achieved, prevention of structural damage is most likely if remission is maintained [7]. Unfortunately, recurrent flares are common in RA patients [8–10]. Furthermore, there is evidence that progression of joint damage may proceed despite the absence of clinical synovitis—that is, in patients with MDA or in remission—presumably due to the presence of subclinical disease activity [11, 12]. In contrast to conventional X-ray scanning, advanced imaging techniques such as magnetic resonance imaging (MRI), ultrasound (US), and positron emission tomography (PET) allow detection and quantification of subclinical synovitis [13–16]. MRI and US abnormalities are associated with future radiological damage and flaring in RA (remission) patients [12, 15–20], but this association is not very strong [21], leaving room for alternative imaging techniques that could further contribute to specificity.

PET depicts biological targets and can be used for sensitive detection of inflammation at molecular and cellular levels. Macrophage-targeting PET tracers, such as ^11^C-(*R*)*-*PK11195 (1-(2-chlorophenyl)-*N*-methyl-*N*-(1-methylpropyl)-3-isoquinoline carboxamide), can visualize inflammatory processes. We have recently shown that PET and macrophage targeting is a promising technique for identification of longstanding RA patients with signs of subclinical synovitis related to short-term flare [15], but such studies have not yet been conducted in early RA patients.

We explored whether ^11^C-(*R*)-PK11195 PET could depict residual disease activity in early RA patients that achieved a state of MDA with intensive DMARD combination treatment. Patients were also followed to determine whether such residual disease activity could be linked to development of flare. Finally, PET results were compared with results from contrast-enhanced MRI.

## Methods

### Patients and study protocol

Patients who were diagnosed with RA (according to the 1987 American College of Rheumatology (ACR) criteria, mean (interquartile range (IQR)) disease duration 9.0 (7.0-15.0) months) years) by rheumatologists at the VU University Medical Center or Jan van Breemen Research institute | Reade were treated according to an intensive regimen in a treat-to-target design, with either COBRA (MTX, SSZ, and initially high-dose prednisone 60 mg per day) or COBRA-light therapy (with MTX up to 25 mg per week and prednisone initially 30 mg per day), in which the therapeutic regimens have recently been shown to be equally effective [5, 6]. Patients were consecutively asked to participate in the current PET substudy if they were classified as having “minimal disease activity without apparent synovitis” (MDA), which was defined as: Disease Activity Score (DAS) in 44 joints <1.6 (which conforms with the outcome definitions of the COBRA-light trial); no tender joints according to the Ritchie articular index (53 joints); and no swollen joints according to a swollen joint count (44 joints) as scored by a trained research nurse (at *T* = 26, 32, 52, or 78 weeks of the COBRA-light trial). Eligibility criteria for the trial can be found in the trial report [5, 6]. Specific criteria for exclusion from this study were the presence of a pacemaker, use of a benzodiazepine agonist 10 days prior to PET scanning, and previous exposure to radioactivity with a yearly cumulative dose of ≥5 mSv. Patients were allowed to continue with DMARD treatment (COBRA, *n* = 11; COBRA-light, *n* = 14) according to the study trial protocol. High (spatial) resolution (*R*)*-*^11^C-PK11195 PET (*n* = 25) and contrast-enhanced MRI (*n* = 24) were performed on both hands and wrists.

Clinical follow-up data up to 1 year from inclusion in this study were retrieved from the trial dataset and included the World Health Organization–International League of Associations for Rheumatology (WHO-ILAR) core set [22], including swollen joint count (44 joints), level of erythrocyte sedimentation rate (ESR), C-reactive protein (CRP) levels, and evaluation of patient and physician global health score by a visual analog scale (VAS, 0–100 mm).

Ethical approval was obtained from the ethics committee of the VU University Medical Center and informed consent was given by all patients prior to inclusion.

### Clinical outcome measures

Flare was defined as the occurrence of at least one swollen joint during a 44-joint examination [23].

The time to follow-up and the number of visits were dependent on the time point within the COBRA-light trial at which patients were eligible for inclusion in the substudy. At least two visits were available for each patient at a median of 9 (IQR 7–15) weeks and 24 (IQR 21–47) weeks. An additional visit was available at 50 (IQR 47–54) weeks and 72 (IQR 70–76) weeks for respectively 18 and 12 patients. For all patients, a visit approximately 52 weeks after inclusion in this substudy (range 45–69 weeks) was available. For investigation of the relationship between the level of cumulative PET/MRI scores and development of flare in time, the cumulative PET and MRI scores of patients who developed a flare early (at 9 weeks, i.e., the median of the first follow-up visit) were compared with those of patients who developed a flare later during follow up (at 24 weeks, i.e., the median of the second follow-up visit).

### PET protocol and data analysis

At baseline, a double-layer ECAT High Resolution Research Tomograph (CTI/Siemens) performed the ^11^C-(*R*)-PK11195 PET scan (total duration: 27 min) of the left and right metacarpophalangeal (MCP), proximal interphalangeal (PIP), and wrist joints (in total, 22 joints per patient) as described previously [14, 15]. Final consensus scores reached by the two observers (CJvdL and OSH), who were blinded to clinical and MRI data, were the input for analysis. Joint uptake and background uptake were semi-quantitatively scored as 0 (absent), 1 (faint), 2 (moderate), or 3 (intense) as described previously [14]. Final scores were calculated per joint by subtracting background scores from joint scores. Joints were considered positive if they had a final score of at least “1”. This cutoff value was chosen based on previous PET results for healthy controls, which were all scored negative after correction for background (i.e., final score 0). Patients were classified as positive if they had at least one PET-positive joint. Per patient, a cumulative PET score (range 0–66) was obtained by summing final individual joint scores of both hands and wrists.

### MRI protocol and data analysis

A 1.5 T whole-body MR scanner (Siemens Sonata, Erlangen, Germany) acquired images of both hand and wrist joints according to OMERACT (Outcome Measures in Rheumatology) guidelines [15, 24]. For MRI, the same joints (all MCP, PIP, and both wrist joints) as for PET were imaged and scored for the presence of synovitis and bone marrow edema on a semi-quantitative 0–3 scale, according to the OMERACT RA MRI Scoring (RAMRIS) system [24]. Consequently, our scoring method included the validated RAMRIS score of the dominant hand, but was expanded by additional scoring for synovitis and (proximal/distal) bone marrow edema of MCP 1 and PIP 1–5 joints of the dominant hand and similar joints of the nondominant hand. Joints were considered positive if synovitis or bone edema were scored at least “1”. Patients were classified as positive if they had at least one positive joint. Two observers (NA and CD) read all scans, blinded to clinical and PET data, and blinded to sequence. If joint scores between observers diverged by ≥2 points, consensus was reached in a separate scoring session. For all other joints, the mean score of both observers was calculated and used for analysis. Per patient, a cumulative MRI score (range 0–288) was calculated by summing all individual (mean) synovitis plus bone marrow edema joint scores of both hands and wrists.

### Statistical analysis

The intraclass correlation coefficient (ICC) (95 % confidence interval (CI)) between two observers was calculated for both PET and MRI cumulative scores.

Differences in cumulative scores between groups with versus without a flare were evaluated with Mann–Whitney *U* tests. *p* <0.05 was regarded as statistically significant.

Point estimates and distribution of data are reported as median (IQR) or mean (standard deviation (SD)). All statistical tests were performed with IBM SPSS statistics 20 (IBM Corp., Armonk, NY, USA).

## Results

### Baseline patient characteristics

Twenty-five RA patients with MDA were included in this study. Patient characteristics are presented in Table 1. All included patients had a DAS in 44 joints <1.6. At the time of inclusion in the present study, the 2011 ACR/European League Against Rheumatism (EULAR) Boolean remission criteria were not yet published. In retrospect, 18/25 (72 %) of patients fulfilled these criteria [25]. For all remaining patients a VAS general health score >10 mm (range 21–77) was the failing criterion. There were no statistically significant differences in patient characteristics between patients with and without a flare (results not shown).

**Table 1:** Patient characteristics

Baseline characteristic	Value
Female	13 (52 %)
Age (years)	53 (13)
Disease duration (months)	9.0 (7.0–15.0)
ESR (mm/hour)	5.0 (2.0–7.5)
CRP (mg/L)	2.5 (1.0–5.0)
VAS general health (0–100 mm)	5.0 (1.0–24.5)
Tender joint count	0
Swollen joint count	0
DAS in 44 joints	0.7 (0.3)
2011 Boolean ACR/EULAR remission	18 (72 %)
ACPA-positive	18 (72 %)
RF-positive	16 (64 %)
Medication	
MTX	12 (48 %)
MTX + SSZ	8 (32 %)
LFL	1 (4 %)
MTX + etanercept	2 (8 %)
MTX + SSZ + etanercept	2 (8 %)
Prednisolone (maximum 7.5 mg per day)	16 (64 %)

Data presented as number (%), mean (standard deviation), or median (interquartile range). *n* = 25

*ACR* American College of Rheumatology; *CRP* C-reactive protein, *DAS* Disease Activity Score, *EULAR* European League Against Rheumatism, *LFL* leflunomide, *MTX* methotrexate, *RF* rheumatoid factor; *SSZ* sulfasalazine, *VAS* visual analog scale

### Baseline PET evidence of subclinical inflammation

**Fig. 1:**
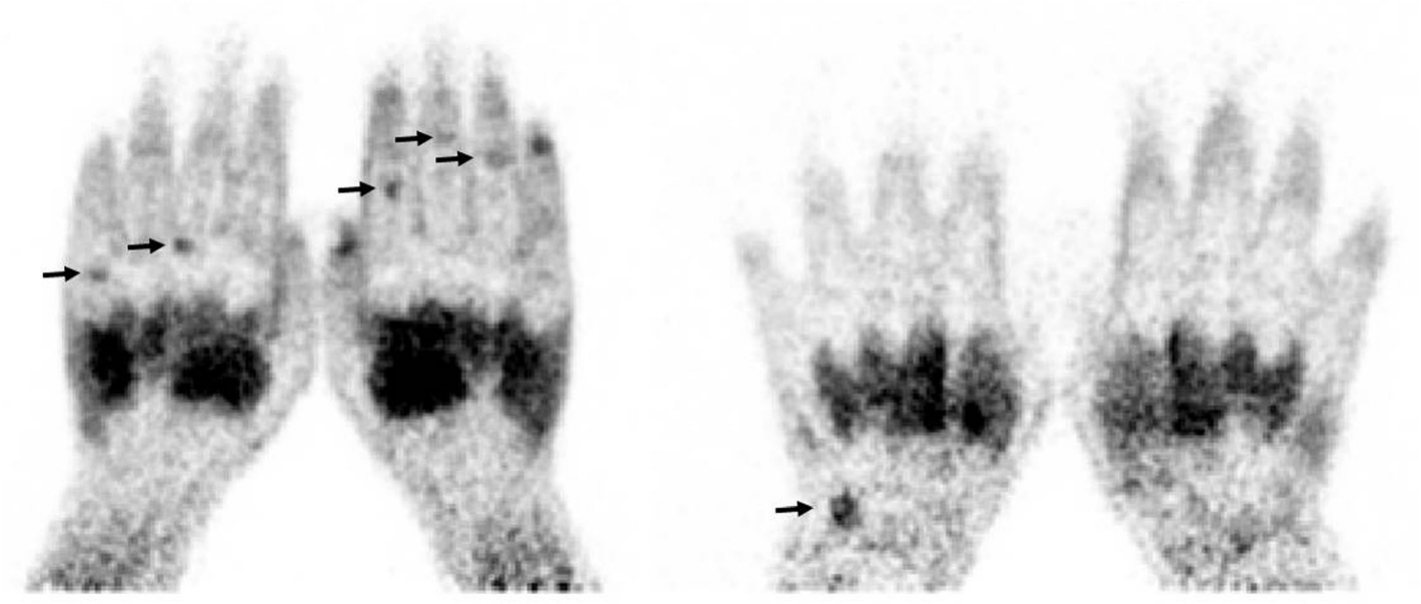
^11^C-(*R*)-PK11195 PET scan. Coronal ^11^C-(*R*)-PK11195 PET scans of two RA patients without apparent synovitis. Enhanced uptake of the macrophage-targeting PET tracer ^11^C-(*R*)-PK11195 is visible as black hotspots (*arrows*). Interosseus muscles and nailbeds show normal background uptake

The reliability between both observers was good (ICC 0.78 (95 % CI 0.57–0.90)). Eleven of 25 patients (44 %) were PET-positive (for a representative PET scan, see Fig. 1), with median 0.0 (IQR 0.0–2.5) positive joints. Median cumulative PET score was 0.0 (IQR 0–2.5, maximum 9). At the joint level, 32/548 (6 %) joints were PET-positive. The most frequent positive PET score was 1; a score of 2 was found in one joint in each of 3/11 (27 %) positive PET scans, and no joints scored 3.

### PET outcome related to development of flare

**Fig. 2:**
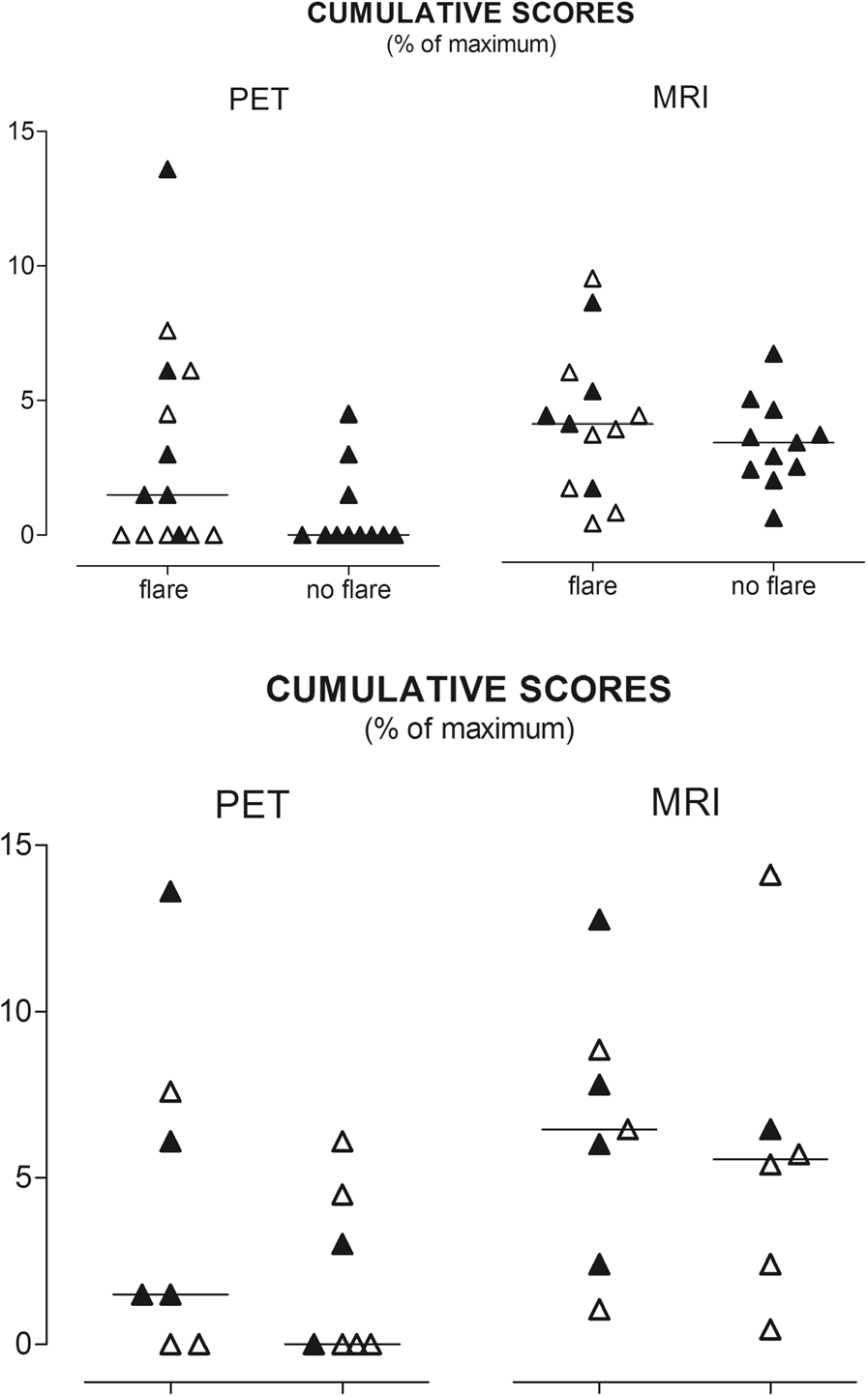
Cumulative PET and MRI scores of patients with and without a flare. *Horizontal line*, median. Scores expressed as a percentage of the maximum possible score (PET, 66; MRI, 288). In patients with a flare, *open symbols* indicate those with a flare outside the hand or wrist. *MRI* magnetic resonance imaging, *PET* positron emission tomography

Fourteen of our patients (56 %) developed a flare within 1 year: this comprised eight of the 11 PET-positive patients and six of the 14 PET-negative patients. Six patients flared in the hands or wrists (the scan region), and five were PET-positive. Only one patient thus had a truly “false-negative” PET in that the flare occurred within the scan region showing no PET signal. The same pattern was seen in the cumulative PET scores: median scores were significantly higher in patients with a flare in the hands/wrists (*n* = 6) than in patients without (*n* = 11): 1.5 (IQR 0.8–5.3) versus 0.0 (IQR 0.0–1.0), *p* = 0.04. Significance was lost in the comparison of all patients with a flare versus those without: median score 1.0 (IQR 0.0–4.0) versus 0.0 (IQR 0.0–1.0)), *p* = 0.10 (Fig. 2, left panel). Results were similar when the definition of flare was modified to “the presence of two swollen joints”, instead of one swollen joint (results not shown).

The relationship between time to clinical flare and the PET result was investigated by comparing cumulative PET scores from the first follow-up visit (median 9 weeks) with those from the second follow-up visit (median 24 weeks). There was a weak trend for an inverse association between cumulative PET scores and time to clinical flare (1.50 (IQR 0.0–7.6) versus 0.0 (IQR 0.0–3.8), *p* = 0.23, respectively; results not shown).

### PET in relation to MRI

MRI scans of 24 patients were available for analysis. The median time interval between PET and MRI was 5 days (IQR 5–5). MRI for one patient failed due to movement artifacts (PET for this patient was negative, flare was observed at 53 weeks after inclusion). The interobserver reliability was excellent with an ICC of 0.94 (95 % CI 0.87–0.97).

The median cumulative score of all MRI scans was 10 (IQR 6–14). All MRI scans were scored positive. Therefore, all PET-positive patients (*n* = 11) also had positive MRI results. Vice versa, 13/24 (54 %) patients with a positive MRI scan had negative PET scans. Bone marrow edema was observed in 11/24 (46 %) patients. No difference in median cumulative MRI scores was found between patients with and without a flare (11.5 (IQR 4.5–16.0) versus 9.5 (IQR 6.5–13.0), *p* = 0.47) (Fig. 2 right panel), and also when the comparison was limited to patients with flare in hands/wrists (*p* = 0.27). Median cumulative MRI scores of patients with a flare at the first follow-up visit (median 9 weeks; 4.0 (IQR 1.6–5.9)) were not significantly different from those with a flare at the second follow-up visit (median 24 weeks; 3.6 (IQR 1.0–4.3), *p* = 0.37) (results not shown).

## Discussion

This novel study demonstrated enhanced uptake of the macrophage PET tracer ^11^C-(*R*)-PK11195 in the hands and/or wrists of almost one-half of a cohort of early RA patients in MDA after intensive combined DMARD therapy. Furthermore, significantly higher cumulative PET scores were observed in the subset of patients that flared in hands/wrists compared with those patients without a flare. These results support our previous results with ^11^C-(*R*)-PK11195 PET in treated RA patients with longstanding disease [15], and suggest that subclinical macrophage activity can be present in treated RA patients with clinically quiescent disease, regardless of disease duration. Because the presence of subclinical disease activity and frequent flares have negative impact on clinical outcome and prognosis, our findings strengthen the case for additional imaging to determine the prognosis of RA patients with MDA without clinically apparent synovitis. It is promising that PET could even distinguish between patients with and without a flare in a setting of optimal suppression of disease activity in a treat-to-target study.

Our MRI results support the findings of several other studies that report MRI synovitis and bone marrow edema—both regarded as signs of disease activity—in up to 96 % and 46 % of RA patients with MDA, respectively [13, 26, 27]. MRI is claimed to be particularly useful in the detection of subclinical disease activity because of its high sensitivity, which enables depiction of subtle signs of inflammation that may not be detected by clinical examination. However, some of these MRI abnormalities may have no clinical relevance, resulting in a low specificity and the need for cutoff levels of diagnostic significance of MRI findings [21]. Our study suggests higher specificity for macrophage-targeted PET both for dichotomous outcome (PET and MRI positivity at a patient level: yes/no) and for the cumulative score. The diagnostic test value of PET could be further increased if the scan region was expanded from the hands to the whole body, for which the PET technique is particularly suited. This was demonstrated in the current study by a significant improvement in differences in cumulative PET scores between the flare and no flare subgroups and an increase from 57 % to 89 % in negative predictive value if only flare in joints within the field of view of the PET scanner was taken into account.

Unfortunately, our study data did not allow conclusions with regard to PET and MRI findings in relation to radiological outcome due to the very low rates of progression overall as reported in the main study [6]. Another limitation is the variation in follow-up intervals for patients, a consequence of adding this study to a running trial. Nevertheless, a minimum of two visits was available for each patient. Future studies in larger study populations are warranted to further prove (validate) the clinical value of ^11^C-(*R*)-PK11195 PET scanning as a predictive tool for flare in RA before it can be implemented in clinical practice.

## Conclusions

This study suggests a potential role for macrophage PET scanning, but not MRI, in early RA patients under intensive treatment to detect subclinical synovitis that may develop into a clinical flare.

## Abbreviations

ACR: American College of Rheumatology
CI: Confidence interval
COBRA: Combinatietherapie bij Reumatoïde Artritis
CRP: C-reactive protein
DAS: Disease Activity Score
DMARD: Disease-modifying anti-rheumatic drug
ESR: Erythrocyte sedimentation rate
EULAR: European League Against Rheumatism
ICC: Intraclass correlation coefficient
IQR: Interquartile range
MCP: Metacarpophalangeal
MDA: Minimal disease activity
MRI: Magnetic resonance imaging
MTX: Methotrexate
PET: Positron emission tomography
PIP: Proximal interphalangeal
RA: Rheumatoid arthritis
RAMRIS: RA MRI Scoring
SD: Standard deviation
SSZ: Sulfasalazine
US: Ultrasound
VAS: Visual analog scale
WHO–ILAR: World Health Organization–International League of Associations for Rheumatology
